# Impact of Tissue Decay on Drying Kinetics, Moisture Diffusivity, and Microstructure of Bell Pepper and Strawberry

**DOI:** 10.3390/foods14193401

**Published:** 2025-10-01

**Authors:** Sindy Palma-Salgado, Luis Vargas, Taha M. Rababah, Hao Feng

**Affiliations:** 1 Department of Food Science and Human Nutrition, University of Illinois at Urbana-Champaign, Urbana, IL 61801, USA; 2 Department of Nutrition and Food Technology, Jordan University of Science and Technology, Irbid 22110, Jordan; 3 Urban and Community Food Complex, College of Agriculture and Environmental Sciences, North Carolina A&T State University, Greensboro, NC 27411, USA

**Keywords:** food waste, thermogravimetric analysis, hot air drying, moisture diffusivity, strawberry, bell pepper

## Abstract

This study investigates the potential to transform plant-based waste into a sustainable resource for animal feed through dehydration. Currently, research on the drying performance of decayed plant tissues remains scarce. To address this gap, we explored the use of a thermogravimetric analyzer (TGA) as a precisely controlled convective drying method to evaluate the drying performance of decayed strawberries (ST) and bell peppers (BP), as models for high- and low-porous structures, respectively. Drying curves, moisture diffusivity, yeast and mold load, and microstructure of decayed plant tissues were evaluated. Our results showed that decayed BP and ST tissues dried up to 22% faster than fresh tissues, with a higher effective moisture diffusivity. Significantly higher yeast and mold counts (log CFU/g) were detected in decayed tissues, resulting in softening and deterioration of the plant tissues. Significant differences were found in the effective moisture diffusivity (D_eff_) of bell pepper (BP) and strawberry (ST), with ST tissues exhibiting a greater degree of decay. The microstructural changes in the cell wall caused by decay influenced drying performance and mass transport kinetics, indicating that drying decayed plant tissues is less time-consuming than drying fresh food. These findings offer critical insights for designing drying processes that enhance the value of food waste.

## 1. Introduction

Food waste from spoilage and consumer rejection is a major and growing issue in the U.S., spanning the food supply chain and resulting in significant losses of food, energy, and water [1,2]. Annually, approximately 1.05 billion tons of food waste are generated, with 60% ending up in landfills [2,3]. Waste arises predominantly from households, supermarkets, food manufacturers, and institutions [4]. Although there are currently no federal legislative mandates for food waste disposal in the U.S., states such as California have introduced measures such as SB 1383, requiring organic waste recycling and 20% food recovery by 2025 [5]. Nationally, the USDA and EPA established a strategic objective in 2015 to reduce food waste by 50% by 2030 [6].

Currently, the methods used for recycling food waste include anaerobic digestion, composting, and diversion to animal feed [7,8]. In the U.S., approximately 10% of surplus food, primarily from manufacturing and grocery stores, is used as animal feed [9]. Similarly, countries such as Korea and Japan reportedly utilize up to 40% of their food waste for this purpose [10]. Recycling food waste can provide multiple benefits. For example, use as animal feed reduces feed costs and environmental impact, while other recycling pathways—such as anaerobic digestion or composting—can generate useful byproducts like biogas and compost [11]. However, challenges include nutrient variability, microbial contamination risks from spoilage and fermentation, and supply chain inconsistencies [12]. Therefore, safe recycling is critical, which can be achieved through controlled heat treatment processes that eliminate pathogens [13].

Food waste often contains high moisture levels (commonly 60–80%), which makes transportation costly and recycling economically challenging [14]. Drying is a practical route to stabilize fruit and vegetable waste (FVW) for animal feed and other value-added uses. By reducing moisture content in FVW, the shelf life of dried products can be extended due to a decreased water activity. During hot-air drying, thermal energy drives the phase change of water into vapor, which is subsequently removed by the airflow [15]. However, this process is highly energy-intensive because of the latent heat required to be stored in water for phase change and the reduced efficiency of heat and mass transfer at low product moisture levels [16]. Optimizing drying processes is crucial, given the typically low profit margins of food waste products. Precise control of drying conditions is necessary to accurately determine drying kinetics; however, many industrial and bench-scale dryers suffer from uncontrolled or poorly measured boundary conditions (air velocity, temperature, humidity, and load variability), making it difficult to isolate true material drying behavior. Thermogravimetric analysis (TGA) overcomes these limitations by providing (i) precise thermal control via programmed ramps and isotherms, (ii) continuous, high-resolution gravimetry (μg-level) to track moisture loss in real time, (iii) well-defined gas composition and flow that reduce and control external mass-transfer effects, and (iv) thin-layer samples that minimize internal temperature and moisture gradients. Together, these features enable precise identification of constant-rate versus falling-rate periods for effective moisture transport parameters [17,18]. For heterogeneous, partially decayed FVW, where tissue breakdown alters water binding and surface barriers, TGA supplies reproducible, mechanistic drying kinetics that can be translated into operating windows for hot-air drying.

Despite the importance of drying FVW, systematic data on the drying characteristics of these decaying food materials remain scarce, leaving processors without evidence-based guidance to select temperatures, air velocities, and residence times in drying system design. This study addresses that gap by providing a comparative evaluation of the drying characteristics of decaying fresh produce, an area that has received little attention, with contrasting external and internal structures and a precisely controlled thermogravimetric method. We selected strawberries (porous surface, soft parenchyma) and green bell peppers (thick, waxy cuticle and firmer pericarp) as model systems to represent distinct tissue structures encountered in FVW streams.

## 2. Materials and Methods

### 2.1. Sample Preparation

Fresh bell peppers (BP) and strawberries (ST) were purchased from a local supermarket and used within 2 h of purchase. Samples were rinsed with distilled water to remove debris, pat-dried using Kimwipes (Kimberly Clark, Irving, TX, USA), and held at 25 ± 1 °C for 30 min inside a laminar flow enclosure (Labconco^®^, Kansas City, MO, USA) operating at 80 fpm (feet per minute) [19] for subsequent analysis.

### 2.2. Fresh Sample Preparation and Characterization

Following the 30 min incubation, approximately 500 g of each sample was prepared for precise drying with a thermogravimetric analyzer and yeast and mold determination. A small piece of each sample (1 ± 0.2 cm^2^) was excised using sterile razor blades (Gillette blade, model 2-010-R3) with a blade thickness of approximately 0.10 mm.

#### 2.2.1. Samples for Thermogravimetric Analyzer Drying Evaluation

Excised sections (1 cm^2^) of BP and ST were used for thermogravimetric analysis. A thermogravimetric analyzer (TGA Q50, TA Instruments, New Castle, DE, USA) was used to determine changes in drying rates and drying curves, following previously published procedures [17], with minor modifications. Since the parenchymatous cells of many plant tissues range from 50 to 300 µm, the 1 cm^2^ sample size used in the TGA tests is regarded as representative for BP and ST in this study. One individual section of the excised sample was placed into the TGA Q50 furnace assembly, and the furnace chamber was closed. The vacuum oven method (24 h at 70 °C, 3.33 kPa) was used to determine the initial moisture content of the fresh and decayed samples, and the equilibrium moisture content (Xe) of the dried samples [20].

#### 2.2.2. Yeast and Mold Load Determination

For mycological analysis, whole fresh BP samples and whole fresh ST (10 g each) were prepared separately by excising square pieces (0.5–1 cm^2^) using a razor blade. Additionally, the calyx of the whole fresh strawberries was excised using a razor blade (Gillette blade, model 2-010-R3) and discarded. For the preparation and plating of inoculum, 10 g of fresh and decayed samples were added to 90 mL peptone water (0.1%) in a stomacher bag. The mixture was homogenized using a stomacher (model 400, Seward Medical, London, UK) for 2 min [21]. Following homogenization, the mixture was allowed to rest for an additional 2 min to allow the incorporation of any foam produced during the high-speed mixing. The homogenate was then serially diluted tenfold, and a 1 mL aliquot of the homogenized samples was inoculated onto the center of 3M^™^ Petrifilm^™^ Rapid Yeast and Mold Count Plates (3M, St. Paul, MN, USA), following the manufacturer’s instructions. The inoculated Petrifilms plates were left to solidify for 15 min and subsequently incubated at 25 °C ± 1 °C in a High-Performance Mechanical Convection Incubator (Thermo Scientific, Waltham, MA, USA). Yeast and mold colonies were manually counted after 3 and 5 days of incubation, respectively. The yeast and mold populations were converted to log_10_ counts and reported as log CFU/g fresh mass. All mycological analyses were performed in triplicate.

### 2.3. Accelerated Decay Sample Preparation

To evaluate the drying performance of decayed strawberries and bell peppers, the second half of the BP and ST samples (about 500 g) was subjected to accelerated decay conditions. Whole strawberries (including leaves and stems) and whole bell peppers (including stems) were placed separately in a single layer in partially opened plastic containers (820 cm^2^, Rubbermaid food storage, Atlanta, GA, USA). To accelerate the rate of naturally occurring decay, a humidity and temperature chamber (Fisher Scientific, Waltham, MA, USA) was set at 25 °C ± 2 °C, with slight modifications to simulate produce shelf life at ambient conditions [22]. A relative humidity of 80% ± 5% was maintained to simulate high humidity conditions that favor the growth of naturally occurring fungal microorganisms residing on the produce surface or within wounds [23]. A BP sample of 10 g was retrieved on day 7 for mycological analysis, following the procedure outlined in Section 2.2.2. ST samples were retrieved from the chamber at days 2, 3, and 4 for thermogravimetric analysis, following the procedure in Section 2.2.1. A ST sample of 10 g was retrieved on day 4 for mycological analysis, following the procedure outlined in Section 2.2.2. The sampling intervals for BP and ST were determined based on preliminary tests and visual observations of the degree of decay.

### 2.4. Precision Drying with a Thermogravimetric Analyzer

A thermogravimetric analyzer (TGA, TA Instruments, New Castle, DE, USA) was used to perform BP and ST drying, following the method of Feng et al. [17] with slight modifications. The BP samples were collected on days 1, 3, 5, and 7 for TGA drying, following the procedure in Section 2.2. Similarly, the fresh and decayed strawberry samples on days 1, 2, 3, and 4 were used for TGA drying. The TGA furnace temperature ramp rate was from 0.1 °C to 200 °C/min until a temperature of 70 °C was achieved, following the method of Feng et al. [17]. Subsequently, the TGA drying was conducted isothermally at 70 °C ± 0.1 °C until a constant weight was achieved when the weight change ≤ 0.01% over 10 min. A built-in thermocouple next to the sample monitored the temperature during the drying experiments. Nitrogen at a rate of 60 mL min^−1^ N_2_ was used as a purge gas to transport heat from the furnace to the sample and remove moisture from the sample chamber. Fresh and decayed BP and ST samples (1 cm^2^) were dried separately. A minimum of six replications was performed for each TGA drying treatment. Thermogravimetric drying data were collected and analyzed using Universal Analysis 2000 software (Version 4.5A, TA Instruments, New Castle, DE, USA). Weight change measurements from the TGA were employed to generate drying and drying rate curves, as well as to estimate the effective moisture diffusivity. It should be noted that in TGA, nitrogen gas is used to carry moisture from the sample surface to the exterior, whereas in real-world drying processes, heated air is typically used for this purpose. However, since the focus of this work is on comparing the moisture removal behavior of fresh and decayed plant tissues, the choice of carrier gas becomes less critical.

### 2.5. Determination of Effective Moisture Diffusivity

The TGA drying curves were analyzed to estimate the effective moisture diffusivity (D_eff_) of fresh and decayed bell pepper and strawberry tissues, employing the slope method [17,24]. For an isotropic slab, Fick’s second law can be written as follows:

(1) $$\frac{\partial X}{\partial t}={𝛻[D}_{eff}𝛻X]=D_{eff}{𝛻}^{2}X$$

In Equation (1), the assumptions include a uniform initial moisture distribution within the sample, negligible external mass transfer resistance, and an isotropic material without shrinkage. The analytical solution to Equation (1) was provided by Crank [25]:

(2) $$X=MR=\frac{X_{t}-\;X_{e}}{X_{0}-X_{e}}=\frac{8}{\pi^{2}}\sum_{n=1}^{\infty }\infty \frac{1}{{\left(2n+1\right)}^{2}}exp\;\left(-{\left(2n+1\right)}^{2}\frac{{\pi^{2}D}_{eff}t}{{4L}^{2}}\right)$$

where MR is dimensionless moisture ratio, *X* is the moisture content in the sample on a dry basis (d.b.), MR is dimensionless moisture ratio, *X_e_* is the equilibrium moisture content (d.b.), *X*_0_ is the initial moisture content (d.b.), *X_t_* is the moisture content at time *t*, D_eff_ is the effective moisture diffusivity (m^2^/s), and *L* is the half-thickness of the sample (m).

For extended drying times, the first term on the right-hand side of Equation (2) becomes dominant [18], thereby reducing Equation (2) to the following:

(3) $$\ln\left(MR\right)=\;\ln\left(\frac{8}{\pi^{2}}\right)-\left(\frac{\pi^{2}D_{eff}}{{4L}^{2}}\right)t$$

When the experimental data are plotted as ln(MR) versus time, a straight line is obtained with a slope of

(4) $$K=-\frac{\pi^{2}D_{eff}}{{4L}^{2}}$$

Then D_eff_ can be determined from the slope using Equation (4). We fitted a straight line on TGA drying curve in SigmaPlot (version 13.0, Grafiti LLC, Palo Alto, CA, USA) to maximize the R^2^ value for the linear section of the curve that we can identify, obtained the slope, and used that slope to estimate D_eff_ values.

### 2.6. Microstructural Characterization of Fresh and Decayed Tissues

The microstructure of the fresh and decayed tissues of strawberries and bell peppers was examined by a scanning electron microscope (SEM) and a Micro CT scan. The samples were first submerged in liquid nitrogen for 5 min in separate containers to freeze the tissues. The frozen samples were then subjected to freeze-drying in a Dura-Dry unit (FTS Systems, Stone Ridge, NY, USA) at −55 °C and 0.1 mbar for 24 h to remove moisture while preserving microstructure [26,27].

Microstructural images of the freeze-dried samples were obtained using an environmental scanning electron microscope (FEI Quanta FEG 450 ESEM, Hillsboro, OR, USA) operated at low vacuum (2 mbar, 20 kV). Bell peppers and strawberries were sliced perpendicularly into sample sections of 1.0 ± 0.2 cm thickness, with the outer surface positioned under the SEM. Three samples at different stages of decay were examined, and ten distinct regions per sample were imaged by moving the samples along the x- and y-axes. Surface changes in both fresh and decayed strawberry and bell pepper tissues were subsequently evaluated.

Micro-CT scanning (µ-CT) was used to compare structural differences between the fresh bell pepper and fresh strawberry tissues, highlighting the contrast between highly porous and low-porosity materials. A 5 cm-long cylindrical sample of fresh BP and ST was placed inside a 100 mL plastic tube to avoid dehydration during the µ-CT scan. A MicroCT system (Xradia MicroXCT-200, Carl Zeiss AG, Germany) was employed to perform multiple scans of fresh bell pepper (BP) and strawberry (ST) tissues. The two-dimensional plane images were reconstructed and rendered into three-dimensional models using a TXM Reconstructor and TXM 3D Viewer software (Workstation 1.2, Carl Zeiss AG, Germany).

### 2.7. Statistical Analysis

The experiments followed a completely randomized design (CRD), with each treatment performed independently at least three times, except for TGA drying, which was performed at least 6 times. Data analysis was carried out using a general linear model in SAS (Version 9.1; SAS Institute, Raleigh, NC, USA) and OriginPro 2016 (OriginLab Corporation, MA, USA). Mean comparisons were conducted using Tukey’s test at a significance level of α = 0.05.

## 3. Results and Discussions

### 3.1. Drying Rates

**Figure 1:**
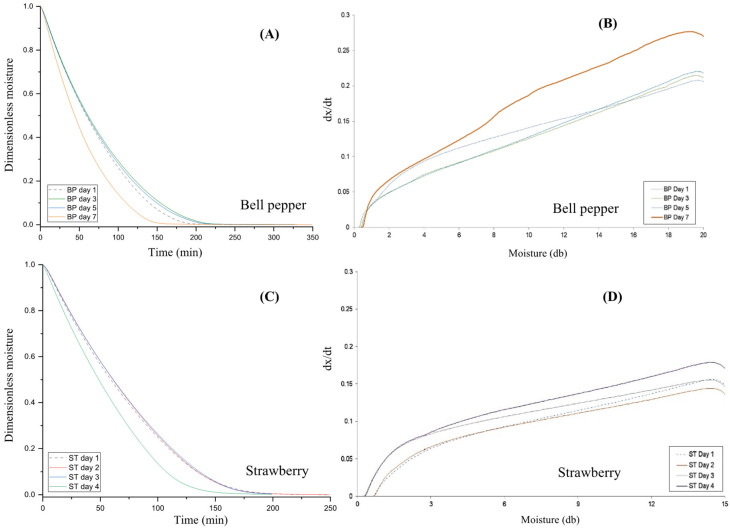
Drying curves (**A**) and drying rate curves (**B**) of bell pepper at 1 (fresh), 3, 5, and 7 days of accelerated decay (25 °C, 80% RH); and drying curves (**C**) and drying rate curves (**D**) of strawberries at 1 (fresh), 2, 3, and 4 days of accelerated decay (25 °C, 80% RH). The unit of dx/dt is (d.b.) min^−1^.

Figure 1A,B show the drying and drying rate curves for fresh bell pepper (BP) samples and those subjected to up to 7 days of accelerated decay. Drying was considered complete when weight loss became negligible. BP samples after 7 days of decay dried approximately 22% faster, reaching constant moisture at 175 min compared with 225 min for fresh samples (Figure 1A). This drying acceleration indicates that tissue degradation may enhance moisture transport, offering valuable insights for optimizing drying strategies in postharvest waste treatment.

Figure 1C,D present the drying curve and drying rate curves for fresh strawberry (ST) (Day 1) and those subjected to up to 4 days of accelerated decay. Similar to the BP drying, strawberries decayed for 4 days reached a constant moisture content about 11% faster than fresh samples (Figure 1C), with negligible weight change occurring at 200 min compared with 225 min. To our knowledge, this is the first report suggesting that moderate decay may facilitate faster drying in strawberries.

With regard to the maximum drying rate of moisture removal with TGA, BP samples after 7 days of accelerated decay exhibited a higher drying rate (dx/dt ≈ 0.27, (d.b.) min^−1^), compared to fresh BP samples (dx/dt ≈ 0.20, (d.b.) min^−1^) (Figure 1B). BP samples subjected to up to 5 days of accelerated decay showed a drying rate similar to that of the fresh samples (Figure 1B). In comparison, strawberry samples after 4 days of accelerated decay had a higher drying rate (dx/dt ≈ 0.18, (d.b.) min^−1^) than both the drying rate of fresh ST samples and those undergoing 2 or 3 days of decay (dx/dt ≈ 0.15, (d.b.) min^−1^) (Figure 1D). The ST samples with up to 2 days of decay exhibited a drying rate similar to that of the fresh samples (Figure 1D).

As vegetable and fruit tissues undergo decay and approach the end of their shelf life, they experience significant structural and physical changes. These alterations are primarily driven by the proliferation of spoilage microorganisms, elevated enzymatic activities, and the degradation of the tissues’ cell wall structures [28,29]. Such transformations can impair the water-binding capacity of the tissues [18], thereby affecting the drying rate of the material. Understanding how tissue decay affects drying kinetics is crucial for designing efficient food waste drying systems, as faster drying rates can lower energy use and better preserve nutritional quality by reducing heat exposure [30].

Drying kinetics of food waste play a key role in shaping effective waste management strategies. Applications include using dried food waste as a partial substitute for animal feed [31], employing drying as a composting pretreatment to cut methane emissions by up to 90% [32], and reducing CO_2_-equivalent emissions by up to 800 kg per 1000 kg of waste diverted from landfills [33]. Drying also adds value by enabling the recovery of bioactive compounds [34].

### 3.2. Effective Moisture Diffusivity

**Figure 2:**
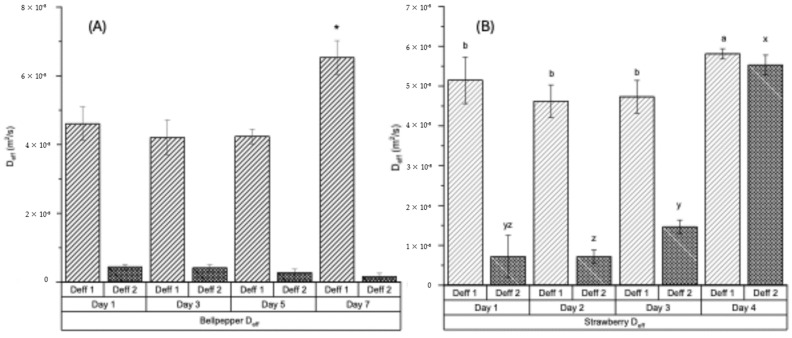
Effective moisture diffusivity (Deff) of bell pepper (**A**) measured at 1 day (fresh) and after 3, 5, and 7 days of accelerated decay (25 °C, 80% RH), and of strawberries (**B**) measured at 1 day (fresh) and after 2, 3, and 4 days of accelerated decay (25 °C, 80% RH). D_eff_ 1 and D_eff_ 2 denote moisture diffusivity for TGA drying in the first falling rate period and second falling rate period, respectively. * Treatment in (**A**) is significantly different (*p* < 0.05). ^a, b, x, y, z^ Treatments in (**B**) labeled with different letters are significantly different (*p* < 0.05).

The drying of BP and ST tissues exhibited a two-phase drying behavior, with two linear sections in the falling rate period identified using the slope method to estimate the effective moisture diffusivity (D_eff_) [17,18]. Therefore, two D_eff_ values were obtained for each produce sample, with D_eff_ 1 representing the moisture diffusivity during the first falling rate period and D_eff_ 2 corresponding to the second falling rate period. The D_eff_ values for fresh and decayed BP are shown in Figure 2A and Figure 2B, respectively. The D_eff_ 1 values ranged from 4.21 × 10^−8^ m^2^/s to 6.53 × 10^−8^ m^2^/s, and D_eff_ 2 values ranged from 0.16 × 10^−8^ m^2^/s to 0.44 × 10^−8.^ m^2^/s (Figure 2A). For strawberries, the D_eff_ 1 ranged from 4.62 × 10^−8^ m^2^/s to 5.81 × 10^−8^ m^2^/s, and D_eff_ 2 from 0.72 × 10^−8^ m^2^/s to 5.53 × 10^−8^ m^2^/s (Figure 2B).

In both produce samples, the drying of decayed tissues is faster compared to the fresh counterparts, as indicated by the significantly higher D_eff_ values for day-7 BP (Figure 2A) and day-4 ST samples (Figure 2B). This trend is evident in the drying curves (Figure 1A,C) and drying rate curves (Figure 1B,D). Tissue degradation in BP and ST may have weakened the binding of water molecules within the biopolymer matrix, thereby facilitating water removal and enhancing both effective moisture diffusivity and mass transfer. A similar increase in moisture diffusivity was reported for tissue pre-treated under ultrasonic wash prior to drying [35]. Effective moisture diffusivity (D_eff_) is a critical parameter in the analysis and design of drying processes. A higher D_eff_ reflects reduced internal resistance to moisture migration toward the tissue surface. This reduced resistance shortens exposure time at elevated temperatures, which in turn improves product quality (by minimizing nutrient degradation) and enhances drying efficiency (through higher throughput and lower energy consumption).

In addition, TGA drying performance differs between the two produce types, as indicated by the distinct D_eff_ values observed during the first and second falling rate periods. In BP, drying during the first falling rate period is considerably faster, as reflected by a higher D_eff_ 1, compared to the second falling rate period (Figure 2A). In contrast, ST maintained relatively high drying rates during the second falling rate period, particularly in the day-4 decayed samples (Figure 2B). The markedly lower D_eff_ 2 in the second falling rate period for BP likely results from its dense, firm structure. Once substantial moisture is removed during the first falling rate period, internal moisture transport becomes increasingly restricted, leading to greater resistance and reduced diffusivity. It should also be noted that the assumptions underlying the application of Fick’s second law in this TGA test may not hold in real-world systems. Therefore, the D_eff_ values obtained here should not be extrapolated to industrial drying processes or heterogeneous product geometries without additional validation.

### 3.3. ESEM Micrographs and Micro-CT Scan

**Figure 3:**
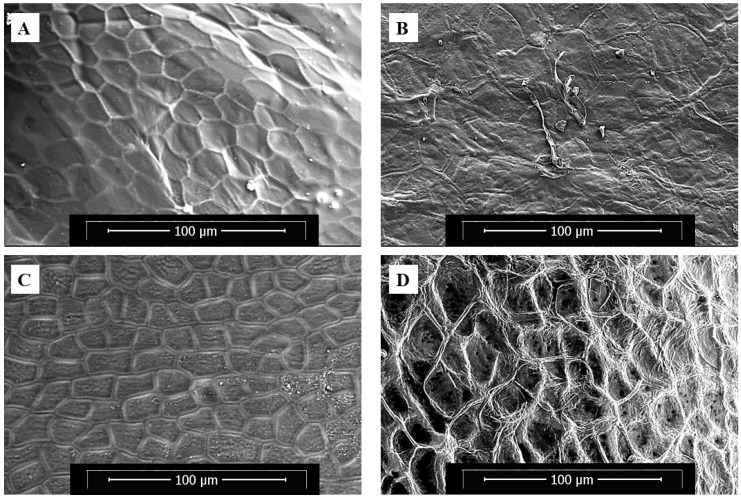
ESEM microimages of strawberry tissues: (**A**) fresh and (**B**) decayed; bell pepper tissues: (**C**) fresh and (**D**) decayed.

The ESEM micrographs of the fresh samples (Figure 3A,C) and the samples undergoing tissue degradation (Figure 3B,D) are shown in Figure 3. Fresh plant cells (Figure 3A,C) exhibit well-preserved morphology with intact, turgid cell walls and closely packed cellular structures. The defined cell boundaries and minimal surface irregularities reflect healthy tissue integrity typical of unspoiled fruit and vegetable samples. In contrast, bell pepper cells after 7 days of decay at 25 °C and 80% RH (Figure 3B) exhibited pronounced morphological degradation. The once well-defined cellular architecture appears collapsed, with extensive cell wall disintegration. These structural changes suggest significant loss of turgor and cellular cohesion, characteristic of advanced decay under high-moisture conditions [28,36]. The ST samples, after 4 days of decay at 25 °C and 80% RH, displayed a different pattern of degradation, characterized by collapsed cell walls and disrupted cellular integrity (Figure 3D). These significant alterations in cell structure align with the observed accelerated drying and elevated D_eff_ in the decayed samples.

**Figure 4:**
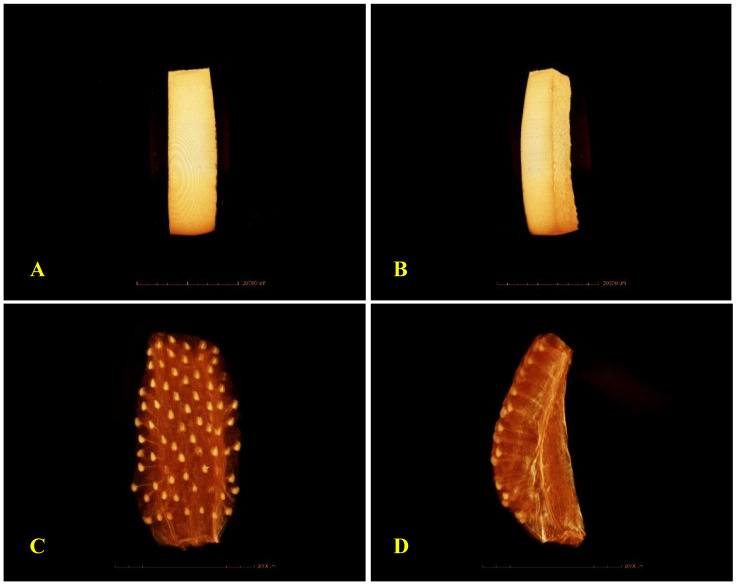
µ-CT scan microimages of external (**A**) and internal (**B**) tissue structure of fresh bell pepper, and external (**C**) and internal (**D**) tissue structure of fresh strawberries.

Figure 4 presents the micro CT Scan (µ-CT) images of fresh BP and ST tissues, highlighting structural differences between the outer tissue of BP (denser skin) and the more porous structure of ST. These images, along with the higher D_eff_ of fresh ST (5.2 × 10^−8^ m^2^/s) compared to fresh BP (4.21 × 10^−8^ m^2^/s), demonstrate the contrasting resistance to mass transfer, with BP serving as a model for low-porosity tissue, and ST for high-porosity tissue, which is also evidenced by the µ-CT images of Prawiranto et al. [37] on microstructure to transport relation in fruits.

### 3.4. Microbial Analysis

To evaluate the progression of spoilage during the accelerated decay assays, yeast and mold populations were quantified at two time points: on day 1 in fresh strawberry and bell pepper samples, and again at the end of the decay period (day 4 for strawberries and day 7 for bell peppers). These measurements provided an indication of microbial activity associated with tissue deterioration. The results of the mycological loads (CFU/g) for fresh BP and ST samples and their decayed counterparts are shown in Table 1.

Compared to the fresh samples, the yeast and mold populations in both the BP and ST samples subjected to accelerated decay were 10 to 10,000 times, or 1 to 4 Log_10_ cycles, higher than those in the fresh samples (Table 1). For example, yeast populations in bell pepper samples increased by 3.5 log CFU/g and mold populations by 4.1 log CFU/g after 7 days of decay compared to fresh samples. Likewise, strawberry samples exhibited a 4.7 log CFU/g rise in yeast counts and a 1.3 log CFU/g rise in mold counts after 4 days of decay compared to their fresh counterparts.

Since no laboratory inoculation of either yeast or molds was performed on the produce samples in this study, the presence of yeast and mold can be attributed to natural sources, such as airborne spores and soil particles [38]. The findings of this study demonstrate that fruit and vegetable spoilage is accompanied by a significant increase in yeast and mold populations. In decayed bell pepper and strawberry samples, tissue softening and cell wall degradation were evident when compared with their fresh counterparts (Figure 3B,D). Pronounced increases in yeast and mold counts were confirmed in the mycological analysis (Table 1). These microorganisms contribute to tissue deterioration by degrading hemicellulose and causing moisture leakage during spoilage [29,39]. The enhanced yeast or mold activities in decayed plant tissues contributed to the destruction of plant cell and tissue integrity, thereby enhancing moisture transport and consequently improving the drying process.

## 4. Conclusions

This study investigated the drying kinetics and transport properties of decayed plant tissues through thermogravimetric analysis (TGA). Decayed bell pepper (BP) and strawberry (ST) samples exhibited drying rates up to 22% higher than their fresh counterparts. Statistically significant differences in the effective moisture diffusivity (D_eff_) were observed for both BP and ST samples across various stages of accelerated decay. Scanning electron microscopy (SEM) revealed distinct microstructural alterations in the decayed tissues, including cell wall degradation. This structural breakdown was corroborated by increased yeast and mold counts in the decayed specimens. Together, these results provide new insight into the drying behavior of decayed plant-based food waste, indicating reduced drying times under comparable operating conditions. These findings can inform the design and optimization of food-waste drying systems, thereby enhancing their technical and economic feasibility.

## Figures and Tables

**Table 1:** Yeast and mold counts (CFU/g) of bell pepper (day 1: fresh; day 7: decayed) and strawberry (day 1: fresh; day 4: decayed) samples.

Sample	Yeast (Mean ± SD log CFU/g)	Mold (Mean ± SD log CFU/g)
Bell pepper day 1	1.3 ± 0.2	ND *
Bell pepper day 7	4.8 ± 0.01	4.1 ± 0.03
Strawberry day 1	ND *	3.6 ± 0.08
Strawberry day 4	4.7 ± 0.36	4.9 ± 0.07

* ND = not detectable.

## Data Availability

Data sharing is not applicable to this article as no datasets were generated or analyzed during the current study.
